# Increasing Access to HIV Testing Through Direct-to-Consumer HIV Self-Test Distribution — United States, March 31, 2020–March 30, 2021

**DOI:** 10.15585/mmwr.mm7038a2

**Published:** 2021-09-24

**Authors:** Jen Hecht, Travis Sanchez, Patrick S. Sullivan, Elizabeth A. DiNenno, Natalie Cramer, Kevin P. Delaney

**Affiliations:** ^1^Building Healthy Online Communities, San Francisco, California; ^2^Emory University, Atlanta, Georgia; ^3^Division of HIV Prevention, National Center for HIV/AIDS, Viral Hepatitis, STD, and TB Prevention, CDC; ^4^NASTAD, Washington, DC.

During 2019, approximately 34,800 new HIV infections occurred in the United States (*1*), and it is estimated that approximately 80% of HIV transmission occurs from persons who either do not know they have HIV infection or are not receiving regular care (*2*). Since 2006, CDC has recommended that persons who are disproportionately affected by HIV (including men who have sex with men [MSM]) should test for HIV at least annually (*3*,*4*). However, data from multiple sources indicate that these recommendations are not being fully implemented (*5*,*6*). TakeMeHome, a novel public-private partnership to deliver HIV self-testing kits to persons seeking HIV testing in the United States, was launched during March 2020 as home care options for testing became increasingly important during the COVID-19 pandemic. The initiation of the program coincided with the national COVID-19 Public Health Emergency declaration, issuance of stay-at-home orders, and other restrictions that led to disruption of traditional HIV testing services. During March 31, 2020–March 30, 2021, 17 state and local health departments participating in the program allowed residents of their jurisdictions to order test kits. Marketing for TakeMeHome focused on reaching gay, bisexual, and MSM through messages and embedded links in gay dating applications. Most participants in the program reported that they had either never tested for HIV (36%) or that they had last tested >1 year before receiving their self-test kit (56%). After receiving the self-test kit, >10% of respondents reported accessing additional prevention services. Health departments can increase options for HIV testing by distributing publicly funded self-test kits to persons without proximate access to clinic-based testing or who prefer to test at home. Increased and regular HIV testing among MSM will help meet annual testing goals.

Self-testing is an effective HIV screening method for MSM (*7*) that can facilitate access to antiretroviral treatment, preexposure prophylaxis (PrEP), and other prevention services (*8*). The COVID-19 pandemic disrupted HIV testing services, persons reported not being able to access HIV testing services (*9*), and hundreds of thousands of HIV screening tests were either delayed or skipped (*10*). CDC sent a “Dear Colleague” letter on April 28, 2020* recommending that grantees consider HIV self-testing as an option to fill the gap in prevention services. This report describes the use and results of TakeMeHome,^†^ a centralized system established during March 2020 to distribute HIV self-test kits.

TakeMeHome offers rapid HIV self-tests (OraQuick In-Home HIV Test), paid for by state and local health departments or other partners at no cost to persons in participating jurisdictions. The program was developed by Building Healthy Online Communities (BHOC)^§^ in partnership with the National Alliance of State and Territorial AIDS Directors (NASTAD)^¶^ and Emory University.** From its inception on March 31, 2020, TakeMeHome established eligibility criteria included residence in a participating zip code, age ≥18 years, and report of no HIV test in the past 12 months. Eligibility was later expanded to persons reporting more recent HIV tests in some participating locations, per jurisdiction request. Several jurisdictions also chose to allow up to two kits per order. All participants were offered a nonincentivized follow-up survey 10 days after their HIV test kit was mailed. This activity was reviewed by CDC and was conducted consistent with applicable federal law and CDC policy. CDC’s role was to provide technical assistance.^††^ Results of the evaluation were analyzed using SPSS software (version 26; IBM).

Characteristics of persons who ordered kits are summarized for all participants. For the subset of persons who ordered kits and responded to the BHOC follow-up survey, reasons for ordering a self-test kit and the proportion of persons who reported accessing other HIV and sexually transmitted infection (STI) prevention services were calculated. To date, two participating health departments established matches between persons who ordered kits and HIV case surveillance to document new diagnoses in persons who had participated in HIV self-testing.

Seventeen health jurisdictions supported self-test kit distribution for their residents during the first year of the program (14 for 6–12 months and three for <6 months). During this time, 5,325 kits were mailed to 4,904 persons. Sixty-seven percent of participants were cisgender men; 6% were transgender, nonbinary, or genderqueer (Table 1). Overall, 1,764 participants (36%) reported never having tested for HIV before ordering an HIV self-test. Among 855 respondents to the follow-up survey (17% of persons who received kits), 73% reported male-to-male sexual contact (Table 2). Most survey respondents reported hearing about the program through marketing by BHOC within gay dating applications (71%), believing that the program addressed issues of convenience (63%) and privacy (46%), and being willing to recommend the program to a friend (90%). After receiving the self-test kit, 10% of respondents reported accessing additional STI testing, and 8% reported accessing PrEP. Among persons who had never previously tested for HIV, 8% reported additional STI testing, and 6% reported accessing PrEP after participating in the program (Figure). The two health departments that matched kit orders to HIV case surveillance estimated that 0.6%–0.8% of those who received a kit were subsequently reported to have newly diagnosed HIV.

**TABLE 1:** Number and percentage of HIV self-test kit orders, overall and by selected characteristics — TakeMeHome program, United States, March 31, 2020–March 30, 2021

Characteristic	No. (%)
**Total number of orders**	4,904 (100)
Sex at birth*	
Male	4,398 (90)
Female	506 (10)
**Current gender identity^†^**	
Man	3,304 (67)
Woman	348 (7)
Transgender/Nonbinary/Genderqueer	282 (6)
Missing	970 (20)
**Age group, yrs**	
18–24	1,461 (30)
25–34	1,907 (39)
35–44	785 (16)
45–54	343 (7)
≥55	147 (3)
Missing	261 (5)
**Race/Ethnicity (multiple responses permitted)** ^§^	
American Indian/Alaskan Native	51 (1)
Asian or Pacific Islander	334 (7)
Black/African American	358 (7)
Hispanic or Latino^¶^	1,295 (26)
Multiple races/Other	170 (3)
White	1,863 (38)
Missing	833 (17)
**Number of sex partners in past 12 months**	
0	7 (<1)
1	1,656 (34)
2	461 (9)
≥3	1,281 (26)
Missing	1,499 (31)
Time since last HIV test**	
Never tested	1,764 (36)
>1 year	2,746 (56)
≤1 year	117 (2)
Missing	277 (6)

* Sex at birth was categorized as male or female. **^†^** Gender identity is based on the participant’s self-categorization in one of three groups at the time of the survey. Thus, man and woman could include persons whose birth sex differs from their current identity. Other gender identities were identified using the umbrella category of transgender/nonbinary/genderqueer regardless of their sex assigned at birth.  ^§^ Race/ethnicity was asked as a single question, and persons could select multiple responses, including “Hispanic or Latino”. If someone selected more than one race and did not select “Hispanic or Latino,” they were categorized as multiracial.  ^¶^ Hispanic/Latino includes anyone who selected this option regardless of whether or not they selected any other race/ethnicity category.

** Time since last HIV test was recorded as “Never,” “Less than 3 months ago,” “4–6 months ago,” “7–12 months ago,” and “More than a year ago,” and recategorized to differentiate those who never tested, tested within the past year, and who last tested >1 year ago. Through March 2021, only four of 17 health jurisdictions participating in TakeMeHome allowed orders from persons who reported testing in the past year.

**TABLE 2:** Follow-up survey results, overall and by selected respondent characteristics — TakeMeHome program, United States, March 31, 2020–March 30, 2021

Characteristic	No. (%)
**Total**	855 (100)
**How did you hear about TakeMeHome?**	
Dating app	605 (71)
Public health campaign	105 (12)
Friend	25 (3)
Google/Other website	97 (11)
Missing	23 (3)
**Risk category (multiple responses permitted)**	
Male-to-male sexual contact	625 (73)
Injection drug use	29 (3)
Partner injection drug use or partner HIV-positive	57 (7)
Multiple sex partners	484 (57)
STI diagnosis or treatment for TB or HCV	60 (7)
No HIV risk reported	85 (10)
Missing	35 (4)
**Reasons for participating (multiple responses permitted)**	
It was free	549 (64)
It was convenient	542 (63)
I prefer testing in the privacy of my home	395 (46)
I feel uncomfortable going to a doctor in my area	267 (31)
I don’t know where to go	160 (19)
COVID-19 has limited regular testing in my area	294 (34)
Missing	40 (5)
**Would you recommend TakeMeHome to a friend?**	
Yes	770 (90)
Maybe	32 (4)
No	6 (1)
Missing	47 (5)

**Abbreviations**: HCV = hepatitis C virus; STI = sexually transmitted infection; TB = tuberculosis.

**FIGURE:**
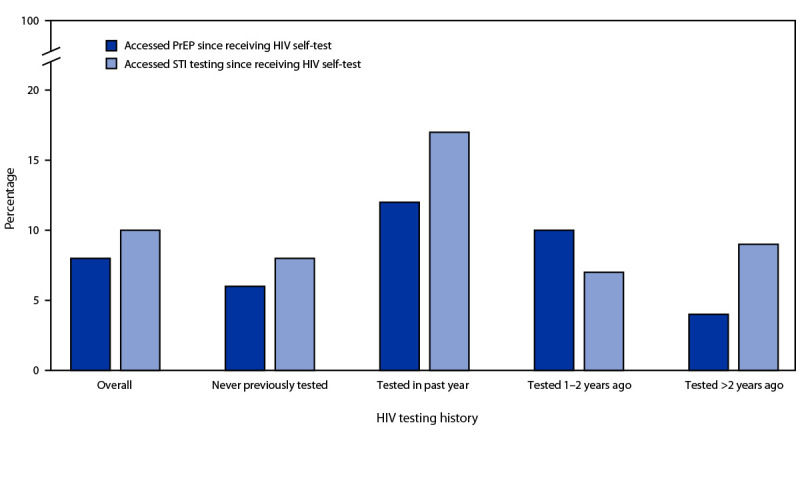
Self-reported access to preexposure prophylaxis or testing for sexually transmitted infection after receiving an HIV self-test kit,* by reported HIV testing history — TakeMeHome HIV self-test kit distribution program, March 31, 2020–March 30, 2021^†^ **Abbreviations**: PrEP = preexposure prophylaxis; STI = sexually transmitted infection. * Among 569 TakeMeHome program participants who responded to a follow-up survey and completed the HIV testing history question. ^†^ As of March 30, 2021, only four of 17 health departments participating in the TakeMeHome program allowed persons who had tested in the past year to order HIV self-test kits; all others required that the participant had not tested in the past year.

## Discussion

TakeMeHome demonstrates the opportunity to provide options for HIV testing to persons who might be reticent or unable to seek clinic- or community-based testing. The program reached critical populations for HIV testing; 36% of participants reported no previous HIV test, and 86% reported recent HIV risk. Most participants stated they would recommend the program to others, and >10% of participants reported that after using the HIV self-test, they sought other HIV and STI prevention services. In addition, 34% of participants reported using TakeMeHome because of decreased availability of HIV testing in their area due to COVID-19, which highlights changing healthcare needs due to the COVID-19 pandemic.

Partnerships between BHOC and dating apps allowed for extensive in-kind promotion of TakeMeHome to specific populations. The program also created social media posts and images to share with jurisdiction partners and provided support for jurisdiction-specific plans to promote the program. States participating in TakeMeHome are also listed on CDC’s HIV self-testing information page,^§§^ and NASTAD and BHOC continue to encourage participation by other jurisdictions. This project also served as a predecessor to a national TakeMeHome demonstration project^¶¶^; the national demonstration project is open to persons aged >17 years living in the United States and Puerto Rico and has partnered with CDC’s Let’s Stop HIV Together Campaign*** to promote the distribution of free HIV self-tests within the most affected populations. The national program model overcame the need to establish separate contractual agreements for each jurisdiction, allowing for nationwide expansion. Through the Let’s Stop HIV Together campaign, self-tests are marketed directly to priority populations using social media, paid media, and partner outreach. The national demonstration project began during February 2021, and through July 2021, a total of 43,568 orders were placed for 76,232 HIV self-test kits by persons from all 50 states, the District of Columbia, and Puerto Rico. 

The findings in this report are subject to at least one limitation. Compared with traditional HIV testing programs, self-testing presents additional challenges to documenting whether the test was used and by whom, as well as challenges documenting a test result and linkage to HIV care or prevention services. TakeMeHome offers multiple resources to help participants interpret their self-test results and access services after testing, but a low response rate for the follow-up survey limited the data available to evaluate accessing of these services and might have introduced bias in the responses. Encouraging participants to access services following a self-test is one method for getting test results reported to public health organizations, but the findings in this report indicate a need to explore other strategies to increase follow-up survey response rates and obtain information about the use of HIV prevention and care after self-testing. For example, HIV prevention and care programs and the HIV surveillance system can document use of HIV self-tests with their clients and among persons with newly diagnosed HIV infection, respectively. 

HIV self-testing is a proven intervention (*7*) that represents a paradigm shift in testing practices and is a key strategy to support the goals of the Ending the HIV Epidemic in the United States initiative (EHE).^†††^TakeMeHome was conceived to help achieve EHE testing goals; the onset of the COVID-19 pandemic accelerated implementation as home health care options became necessary. This report provides data indicating that implementation of Internet-based self-test distribution reached populations of MSM who had never tested or who tested less frequently than annually. Further, HIV self-test distribution addresses many privacy concerns, and this program demonstrated, among the subset who provided follow-up data, that self-testing served as a bridge to additional HIV and STI prevention services for persons who needed them. However, data from this report suggest limited coverage of the program among Black persons. Further expansion to include marketing tailored to minority groups disproportionately affected by HIV, especially Black and Hispanic MSM, as well as engaging health department jurisdictions with higher proportions of disproportionately affected populations will be necessary as the program expands. Local and national public health programs can further expand access to HIV testing through self-testing programs and, through focused advertising, might be able to increase the number of persons tested and testing frequency among MSM and other populations disproportionately affected by HIV to help achieve the goals of EHE.

**Summary**

**What is already known about this topic?**

Gay, bisexual, and other men who have sex with men (MSM) should be tested for HIV at least annually. Major disruptions to HIV testing services occurred during the COVID-19 pandemic.

**What is added by this report?**

During March 2020–March 2021, a novel public-private partnership provided free HIV self-test kits directly to MSM. Most participants reported they had never tested (36%) or tested >1 year ago (56%); approximately 10% reported accessing services including sexually transmitted infection testing and preexposure prophylaxis after using the self-test.

**What are the implications for public health practice?**

Public funding of HIV self-testing can engage MSM who never previously tested and might increase HIV testing frequency among this population.

## Footnotes

*
https://www.cdc.gov/nchhstp/dear_colleague/2020/dcl-042820-HIV-self-testing-guidance.html

†
https://takemehome.org/

§
https://bhocpartners.org/

¶
https://www.nastad.org/domestic/hiv-prevention

**
https://prismhealth.emory.edu/

†† 45 C.F.R. part 46, 21 C.F.R. part 56; 42 U.S.C. Sect. 241(d); 5 U.S.C. Sect. 552a; 44 U.S.C. Sect. 3501 et seq.

§§
https://www.cdc.gov/hiv/basics/hiv-testing/hiv-self-tests.html

¶¶
https://together.takemehome.org

***
https://www.cdc.gov/stophivtogether/index.html

††† TakeMeHome is intended to help mitigate the impact of undiagnosed and untreated HIV by expanding access to testing and treatment nationwide to achieve the goal of diagnosing persons with HIV as early as possible after infection. https://www.hiv.gov/federal-response/ending-the-hiv-epidemic/overview
